# The Role of microRNAs in Cholangiocarcinoma

**DOI:** 10.3390/ijms22147627

**Published:** 2021-07-16

**Authors:** Tingting Shi, Asahiro Morishita, Hideki Kobara, Tsutomu Masaki

**Affiliations:** Department of Gastroenterology and Neurology, Faculty of Medicine, Kagawa University, Kagawa 761-0793, Japan; asahiro@med.kagawa-u.ac.jp (A.M.); kobara@med.kagawa-u.ac.jp (H.K.)

**Keywords:** microRNA, cholangiocarcinoma, diagnostic biomarker, prognostic biomarker

## Abstract

Cholangiocarcinoma (CCA), an aggressive malignancy, is typically diagnosed at an advanced stage. It is associated with dismal 5-year postoperative survival rates, generating an urgent need for prognostic and diagnostic biomarkers. MicroRNAs (miRNAs) are a class of non-coding RNAs that are associated with cancer regulation, including modulation of cell cycle progression, apoptosis, metastasis, angiogenesis, autophagy, therapy resistance, and epithelial–mesenchymal transition. Several miRNAs have been found to be dysregulated in CCA and are associated with CCA-related risk factors. Accumulating studies have indicated that the expression of altered miRNAs could act as oncogenic or suppressor miRNAs in the development and progression of CCA and contribute to clinical diagnosis and prognosis prediction as potential biomarkers. Furthermore, miRNAs and their target genes also contribute to targeted therapy development and aid in the determination of drug resistance mechanisms. This review aims to summarize the roles of miRNAs in the pathogenesis of CCA, their potential use as biomarkers of diagnosis and prognosis, and their utilization as novel therapeutic targets in CCA.

## 1. Introduction

**Figure 1:**
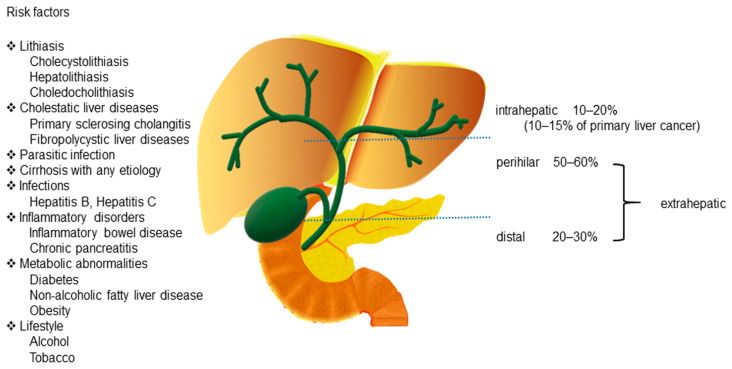
Anatomical classification of cholangiocarcinoma and its risk factors.

Cholangiocarcinoma (CCA) includes a diverse group of biliary epithelial malignancies that involve all points of the biliary tree. Depending on the anatomic location, CCAs are classified into three subtypes: intrahepatic (iCCA), perihilar (pCCA), and distal (dCCA) [1,2]. Among them, pCCA and dCCA are also referred to as “extrahepatic CCA” (eCCA). pCCA, the most common CCA, accounts for 50–60% of all CCAs, followed by dCCA, which accounts for 20–30% of all cases [1]. iCCA is the second most common primary liver cancer after hepatocellular carcinoma (HCC) and accounts for 10–15% of all primary hepatic malignancies [3]. Additionally, a rare type of primary liver cancer, mixed hepatocellular cholangiocarcinoma (HCC-CCA), accounts for <1% of all cases according to the World Health Organization (WHO) [4] (Figure 1).

CCAs are aggressive tumors that account for approximately 3% of all gastrointestinal cancers [5]. CCAs are usually asymptomatic in the early stages and are typically diagnosed at an advanced stage. Although surgery is a therapeutic strategy for patients with CCAs, the 5-year postoperative survival rate (7–20%) remains low because of the challenge of diagnosing patients at an early stage [1,6]. Therefore, developing advanced diagnostic techniques and exploring the mechanisms underlying CCA development and progression can be effective approaches to improve the outcomes for patients with CCA.

**Figure 2:**
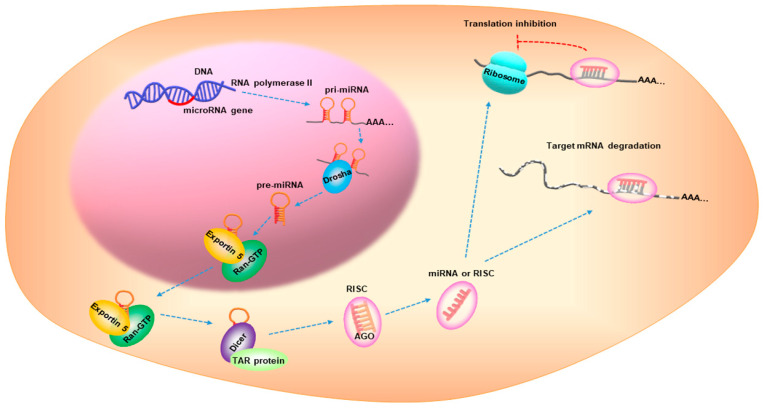
microRNA biogenesis. AGO, Argonaute; RISC, RNA-induced silencing complex.

MicroRNAs (miRNAs) are small, non-coding RNAs with a 17–25 nucleotide length [7]. miRNA biogenesis is a multistep process that is categorized into: transcription, nuclear cropping, export to cytoplasm, and cytoplasmic dicing [8]. miRNA genes are transcribed as primary RNA (pri-miRNA) by RNA polymerase II (pol-II) and are processed by Drosha, a nuclear enzyme of the RNase III family, in the nucleus to release a hairpin-shaped precursor called “pre-miRNA”. Pre-miRNA is recognized by Exportin 5/Ran-GTP transporter and is exported from the nucleus to the cytoplasm. The pre-miRNA is then cleaved by Dicer and the TAR RNA-binding protein to produce a miRNA duplex, which is then loaded onto the Argonaute (AGO) protein to assemble the RNA-induced silencing complex (RISC). One strand remains on the AGO protein to form the mature miRNA, while the other strand is degraded. The mature miRNA represses gene expression by interacting with the complementary sequences in the 3′-untranslated region of the target mRNAs [8,9,10] (Figure 2). Over 5000 miRNAs from diverse organisms are registered in online databases, such as the miRBase (www.mirbase.org, accessed on 9 May 2021). In humans, approximately one-third of the miRNAs are organized in clusters and contain two or more miRNAs with similar sequences [11], possibly leading to combinatorial diversity and synergy in the biological effects of the miRNAs. Furthermore, approximately 30% of the human genes are regulated by miRNAs via signaling pathways [12].

Cancer is a complex genetic disease associated with gene mutations and deregulation of the gene expression. During the last decade, many studies have focused on miRNAs and cancer and have highlighted the impact of miRNAs on gene expression. In this review, we have comprehensively discussed the association between miRNAs and CCA; we have also summarized the roles of miRNAs in the pathogenesis of CCA, their potential use as biomarkers of diagnosis or prognosis, and their possible use as novel therapeutic targets in CCA.

## 2. Epidemiology

The mortality rates of iCCA have increased globally in recent years, with the highest rates reported from 2010 to 2014 (1.5–2.5/100,000 in men and 1.2–1.7/100,000 in women) based on the data of 32 selected countries from the WHO and Pan American Health Organization databases [13]. In addition, in Japan, the mortality rate associated with eCCA is 2.8/100,000 in men and 1.4/100,000 in women [13]. The data from the National Center for Health Statistics between 1999 and 2014 in the USA showed that CCA mortality was 36% higher in patients with age >25 years, and the mortality was lower in females than in males (risk ratio [RR] = 0.78, 95% confidence interval [CI] = 0.77–0.79) [14]. Differences in CCA incidence rates have been reported among different racial and ethnic groups, with the highest rates reported in Southeast Asia and the lowest in Australia [15]. A study in Western Europe indicated that the incidence rates of iCCA increased considerably between 1999 and 2009, especially in the population in the age group of 45–59 years [16]. In contrast, research from the USA has shown that the incidence of iCCA has remained stable from 1992 to 2007; whereas, the incidence of eCCA has been increasing considerably [17]. In Japan, from 1976 to 2013, a total of 14,287 cases of CCA have been identified, and iCCA was more likely to develop in younger patients. The prognosis of iCCA was poorer in comparison to that of eCCA; however, the prognosis of both iCCA and eCCA cases improved after 2006 [18].

## 3. Risk Factors

Although the occurrence and etiology of CCA have not been determined, there are several well-established risk factors associated with chronic biliary epithelium inflammation and cholestasis. These include lithiasis [19]; cholestatic liver diseases, such as primary sclerosing cholangitis (PSC) [20] and fibropolycystic liver diseases [21]; parasitic infections by *Opisthorchis viverrini*, *Clonorchis sinensis*, and *Schistosomiasis japonica* [19,22]; cirrhosis with any etiology [23]; infections, such as hepatitis [23,24]; inflammatory disorders, such as inflammatory bowel disease (IBD) [25] and chronic pancreatitis [25]; metabolic abnormalities [25,26]; and lifestyle [27,28] (Figure 1). 

A Chinese study has indicated that biliary stone diseases, including choledocholithiasis (odds ratio [OR] = 2.704, 95% CI = 1.054–6.941), hepatolithiasis (OR = 3.278, 95% CI = 1.226–8.766), and cholecystolithiasis (OR = 4.499, 95% CI = 2.990–6.769) are risk factors for hilar CCA [19]. Approximately 7% of patients (325 cases) with hepatolithiasis developed CCA as per the data from a nationwide survey in Japan in 2006 [29]. 

PSC, a chronic cholestatic liver disease with an unclear etiology, is characterized by inflammation and fibrosis with multifocal biliary strictures. Additionally, PSC is closely associated with IBD, and about two-thirds of the patients in Northern Europe and the USA have PSC concurrently with IBD [30]. The incidence of CCA in patients with PSC is approximately 0.6–1.5% per year [31]. A genetic study of 186 patients with PSC-biliary tract cancer showed eCCA with high genomic alterations in *TP53* (35.5%), *KRAS* (28.0%), *CDKN2A* (14.5%), and *SMAD4* (11.3%), and even in underlying druggable mutation genes, such as *HER2*/*ERBB2* [32]. Moreover, a 10-year nationwide population-based study from the UK suggested that development of PSC increases the risk of CCAs (hazard ratio [HR] = 28.46, *p* < 0.001) in patients with PSC-IBD, and it also increases the risk of HCC (HR = 21.00, *p* < 0.001), gallbladder cancer (HR = 9.19, *p* < 0.001), pancreatic cancer (HR = 5.26, *p* < 0.001), and colorectal cancer (HR = 2.43, *p* < 0.001) [33]. 

Certain regions of Southeast Asia, such as North and Northeast Thailand, where *Opisthorchis viverrini* infestation is prevalent, show high CCA burden, with 19.3% and 15.7% of the population having the infection, and CCA incidence rates of 14.6/100,000 and 85/100,000, respectively [34,35]. Southeastern and Northeastern China, Korea, Northern Vietnam, and Eastern Russia show a prevalence of human *Clonorchis sinensis* infections [36]. Furthermore, the prevalence of liver fluke (OR = 10.088, 95% CI = 1.085–93.775) is reportedly higher in patients with hilar CCA than in healthy controls [19]. Additionally, there are reports of CCA associated with *Schistosomiasis japonica* infection [37].

Approximately 57% of global cirrhosis cases are induced by chronic infection with hepatitis B (HBV) and hepatitis C viruses (HCV) [38]. Several meta-analyses have indicated that HBV or HCV infection is associated with an increased risk of CCA, especially iCCA [39,40,41]. Cirrhosis, diabetes, and obesity are also risk factors for CCA [41]. A case-control study showed that cirrhosis is a major risk factor for iCCA; other risk factors include nonspecific cirrhosis (adjusted OR = 27.2), HCV infection (adjusted OR = 6.1), diabetes (adjusted OR = 2.0), and alcoholic liver disease (adjusted OR = 7.4) [23]. Furthermore, another study indicated that risk factors associated with iCCA and eCCA were nonspecific cirrhosis, chronic pancreatitis, diabetes, alcoholic liver disease, biliary cirrhosis, and cholelithiasis. Factors associated with iCCA include non-alcoholic fatty liver disease (NAFLD), obesity, and smoking [42].

## 4. The Role of miRNAs in CCA

In recent decades, several studies have focused on the role of miRNAs in cancers. miRNAs play a key role in cancer cell regulation, and are associated with the progression of the cell cycle, apoptosis, metastasis, angiogenesis, glycolysis/Warburg effect, autophagy, therapy resistance, and epithelial–mesenchymal transition (EMT).

### 4.1. miRNAs Associated with CCA Risk Factors

miRNAs play an important role in regulating physiological and pathophysiological functions. In gallstone disease, upregulated miR-210 reduces the expression of its target gene, *ATP11A*, in human gallbladder epithelial cells to regulate the ABC transporter pathway [43]. The pathological mechanism of hepatolithiasis is closely related to chronic inflammation and overexpression of mucin 5AC (MUC5AC). miR-130b inhibits the expression of specificity protein 1 (Sp1), which is followed by a decrease in the expression of MUC5AC [44]. In addition, a clinical control study indicated that the expression levels of miR-21 and miR-221 were upregulated in CCA associated with hepatolithiasis [45]. In PSC, increased melatonin reduces biliary hyperplasia and liver fibrosis by overexpressing arylalkylamine N-acetyltransferase (*AANAT*) in the pineal gland. Moreover, inhibition of miR-200b reduces the expression of fibrotic and angiogenic genes, such as *Col1a1*, *Fn-1*, *Vegf-a*/*c*, *Vegfr-2*/*3*, *Angpt1*/*2*, and *Tie-1*/*2* [46]. In schistosomiasis, miR-21, miR-96, miR-351, miR-146a/b, and miR-27b promote hepatic fibrosis by regulating signaling pathways [47,48]. During liver cirrhosis progression, miR-378 plays a key role in promoting hepatic inflammation and fibrosis via the NF-κB-TNFα axis in non-alcoholic steatohepatitis [49]. Increased miR-30a expression downregulates extracellular matrix-related gene expression, such as that of *α-SMA*, *TIMP-1*, and collagen I, and prevents liver fibrogenesis by directly targeting the Beclin1-mediated autophagy [50]. Activation of hepatic stellate cells (HSCs) is a major step in the initiation and progression of hepatic fibrosis and overexpression of miR-214-3p suppresses the expression of suppressor-of-fused homolog (Sufu) to promote HSC activation and fibrosis development [51]. 

HBV infection induces a spectrum of liver diseases ranging from acute infection to chronic hepatitis, cirrhosis, and HCC [52]. Wang et al. have indicated that miR-98, miR-375, miR-335, miR-199a-5p, and miR-22 are involved in HBV infection [53]. The expression of miR-192-3p is negatively associated with increased levels of HBV DNA in the serum of patients with HBV. HBV induces autophagy to promote its replication by the miR-192-3p-XIAP axis via the NF-κB signaling [54]. Additionally, miR-224 [55] and miR-1231 [56] suppress HBV replication by inhibiting SIRT1-mediated autophagy and targeting the core mRNA, respectively. Other studies have reported that the miR-99 family promotes replication by enhancing autophagy through the mTOR/ULK1 signaling [57]. 

HCV infection is a global health problem that leads to chronic carriage in 70–80% of all cases and presents a high risk of cirrhosis and cancer [58]. miR-215 [59] promotes HCV replication by targeting *TRIM22* and miR-21-5p [60], and it enhances the HCV life cycle and steatosis induced by the viral core 3a protein and other promoters such as miR-122 [61]. Overexpression of miR-199a suppresses HCV genome replication [62], and miR-130a [63] inhibits HCV replication via an Atg5-dependent autophagy pathway. 

Inflammation is associated with cancer origin and is based on an environment rich in inflammatory cells and factors, such as activated stroma and DNA-damage-promoting agents. Moreover, activation of the inflammation signaling pathway enhances cell proliferation [64]. In IBD, miR-301a is overexpressed in peripheral blood mononuclear cells and inflamed mucosa, which promotes mucosal inflammation by inducing IL-17A and TNF-α expression [65]. Increased miR-31 expression in colon tissues of patients with IBD reduces the inflammatory response by inhibiting the expression of IL-7R, IL-17RA, and signaling proteins (GP130). In addition, miR-31 promotes epithelial regeneration via the Wnt and Hippo signaling pathways [66]. In chronic pancreatitis, upregulated miR-15 and miR-16 expression can alleviate apoptosis and fibrosis by targeting both *BCL-2* and *SMAD5* [67].

Diabetes, a metabolic disease, is characterized by high blood sugar and insulin resistance, which are risk factors for HCC and CCA. Various miRNAs have been implicated in the regulation of diabetes [68]. For instance, miR-7 and miR-375 are essential for pancreatic β-cell differentiation and development; miR-184, miR-33, miR-187, miR-29a, and miR-30a are involved in insulin secretion; and miR-15b, miR-199a, miR-181a, and miR-143 are associated with insulin resistance [68]. miR-10b-5p regulates diabetes via the KLF11-KIT pathway [69], and exosomal-derived miR-690 improves insulin sensitivity [70]. NAFLD is caused by triglyceride accumulation, which increases the risk of various liver diseases, such as steatosis, non-alcoholic steatohepatitis, fibrosis, cirrhosis, and ultimately HCC [71]. miR-122 is involved in triglyceride and cholesterol metabolism by targeting the expression of *SREBP-1c*, *SREBP-2*, and *HMGCR*. miR-34a is involved in hepatic lipid metabolism, fatty acid β-oxidation, and apoptotic pathways, and miR-29 is associated with fibrosis [71]. Overexpression of miR-132 alters serum and hepatic lipid profiles, thereby inducing hepatic steatosis [72]. 

Alcohol consumption is closely associated with liver injury, especially alcoholic liver disease. A recent study indicated that alcohol decreases the expression of miR-148a in hepatocytes through FoxO1, thereby facilitating *TXNIP* overexpression and NLRP3 inflammasome activation-induced hepatocyte apoptosis [73]. miR-155 promotes alcohol-induced steatohepatitis and fibrosis [74]. Tobacco smoke induces miR-25-3p maturation via m6A modification, which in turn promotes the development and progression of cancers [75,76] (Table 1).

### 4.2. Dysregulation of miRNAs in CCA

The expression of miRNAs can be correlated with the cancer type and other clinical variables. As each miRNA has multiple target genes, it regulates the target gene expression in a complex manner. This suggests that miRNAs are involved in almost all aspects of cancer biology [7,8,9]. During CCA development, some miRNAs are upregulated, i.e., they act as “oncogenic miRNAs”, whereas, other miRNAs are downregulated and act as “suppressors” (Table 2).

#### 4.2.1. Oncogenic miRNAs in CCA

Upregulated miRNAs in CCA tissues or cells are known as oncogenic miRNAs. Oncogenic miRNAs, such as miR-21 [82] and miR-191 [85], promote CCA cell proliferation, metastasis, angiogenesis, autophagy, and EMT. miR-21 is a well-known oncogenic miRNA that is overexpressed in various cancers such as HCC [117] and breast cancer [118]. In CCA, miR-21 promotes cancer cell proliferation by inhibiting the expression of programmed cell death 4 (PDCD4) and tissue inhibitor of metalloproteinase 3 (TIMP3) [119] by targeting *15-PGDH*/*HPGD* [82]. Moreover, miR-21 promotes iCCA cell growth by targeting *PTPN14* and *PTEN* as its functional targets [120]. A meta-analysis showed that miR-21 expression is associated with poor prognostic outcomes in patients with CCA (HR = 1.88, 95% CI = 1.41–2.51), and it could predict shorter overall survival in patients with CCA [121]. In addition, a recent study indicated that plasma miR-21 and miR-122 levels were significantly higher in patients with iCCA than in controls (healthy, benign liver lesions, other malignant liver tumors), and the increased plasma miR-21 level was correlated with a larger tumor size [122]. Meanwhile, a novel three-marker model comprising plasma miR-21, miR-122, and CA19-9 has been used to increase the diagnostic sensitivity (operating characteristic curve (AUC) = 0.853; 95% CI = 0.824–0.879; sensitivity = 73.0%; specificity = 87.4%) [122].

A meta-analysis suggested that elevated expression levels of miR-21, miR-26a, miR-29a, miR-181c, miR-191, miR-192, miR-200c, and miR-221 are associated with poor survival in patients with CCA; whereas, decreased expression of miR-34a, miR-106a, miR-203, and miR-373 are associated with poor prognosis [121]. Li et al. [85] demonstrated that miR-191 was significantly overexpressed in iCCA tissues, and it promoted cell proliferation, invasion, and migration by targeting *TET1* both in vitro and in vivo. Additionally, miR-191 is an independent risk factor for poor prognosis in patients with iCCA (HR = 3.742, 95% CI = 2.080–6.733). Further, miR-181c promotes CCA cell proliferation, chemoresistance, and metastasis by targeting *NDRG2*, and overexpression of miR-181c is associated with poor overall survival in patients with CCA [86]. 

#### 4.2.2. Tumor Suppressor miRNAs in CCA

The association of tumor suppressor miRNAs, such as miR-34a [91], miR-122 [102], miR-22 [105], and miR-101 [108], is well established in many cancer types. miR-34a expression is often decreased in cancers. It is transcriptionally controlled by *TP53* and regulated by multiple p53-independent mechanisms. miR-34a, a candidate tumor suppressor miRNA, regulates multiple targets, such as *MYC*, *MET*, *CDK4*/*6*, *NOTCH1*, *NOTCH2*, *BCL2*, and *CD44*, all of which have been implicated in tumorigenesis and cancer progression [123]. 

miR-122 is a tumor suppressor in various cancer types, including CCA. It inhibits proliferation and metastasis by targeting *ALDOA* [103] and chloride intracellular channel 1 (*CLIC1*) [124]. Moreover, miR-122 is a regulator in various liver diseases, including HCC [125].

miR-22 plays an important role in many cancer types and has been shown to modulate some oncogenic processes, such as proliferation, apoptosis, angiogenesis, immune response, and metastasis [105]. In an in vitro study on CCA, overexpression of miR-433 and miR-22 was demonstrated to suppress cell proliferation and cellular migration by targeting *HDAC6* [105]. In addition, a survival analysis indicated that *DEPDC1*, *FUT4*, *MDK*, *PACS1*, *PIWIL4*, miR-22, miR-551b, and cg27362525 and cg26597242 CpGs can be used as prognostic markers of CCA. Although miR-22 is a known tumor suppressor, its high expression is correlated with poor survival of patients with CCA [126].

**Figure 3:**
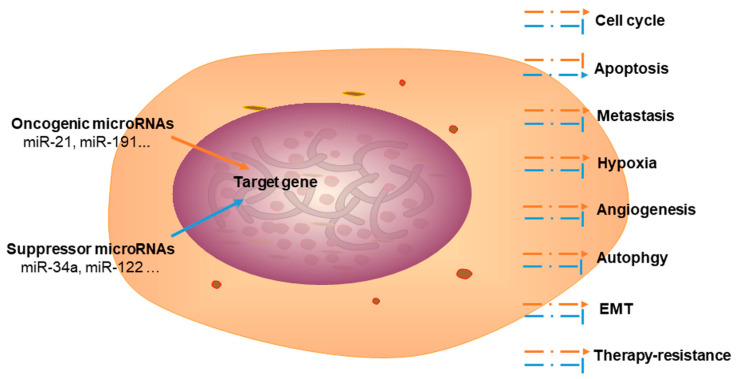
The functions of oncogenic and suppressor microRNAs in cholangiocarcinoma. EMT, epithelial–mesenchymal transition.

miR-101 has been shown to be a tumor suppressor in certain cancers. For instance, miR-101 overexpression considerably inhibits CCA cell proliferation and angiogenesis by targeting vascular endothelial growth factor (*VEGF*), cyclooxygenase-2 (*COX-2*) [108], and *E2F8* [109]. *EZH2* is also a target gene of miR-101 that regulates CCA cell proliferation [127] (Figure 3).

### 4.3. miRNAs as Biomarkers for CCA

CCA is commonly diagnosed through a combination of clinical details, biochemical information, radiological imaging, and histology. Histology is usually considered as the “golden standard” to confirm a diagnosis. Radiological imaging techniques, such as ultrasound, computed tomography, magnetic resonance imaging/magnetic resonance cholangiopancreatography, and positron emission tomography, have been used to diagnose CCA subtypes. 

Carbohydrate antigen 19-9 (CA19-9), a non-specific tumor biomarker, helps in the diagnosis of CCA; however, this biomarker lacks sensitivity and specificity, particularly in the early stages of CCA [2]. Most patients with early-stage CCA are usually asymptomatic, and are thus, diagnosed at an advanced stage. Tumor biomarkers have been widely used to improve early-stage diagnosis and prognosis prediction. In recent years, many studies have examined miRNAs as potential biomarkers for the early diagnosis and prognosis prediction in case of CCA (Table 3).

Numerous studies have reported the use of miRNAs as biomarkers in CCA. miR-21, an oncogenic miRNA, [82], is a potential biomarker for both diagnosis [45] and prognosis [121]. In addition, miR-21 and miR-221 aid in the diagnosis of CCA associated with hepatolithiasis with an increased accuracy (AUC = 0.911) and a sensitivity and specificity of 77.42% and 97.50%, respectively [45]. Serum miR-26a is upregulated in patients with CCA, and its expression levels are associated with the tumor-node-metastasis stage. miR-26a can be clinically beneficial as a diagnostic biomarker for CCA, as it has previously shown an AUC = 0.899, 84.8% sensitivity and 81.8% specificity [128]. Moreover, high expression of serum miR-26a is also an independent predictor of poor overall (HR = 3.461, 95% CI = 1.331–5.364) and progression-free survival in patients with CCA (HR = 4.226, 95% CI = 1.415–10.321) [128]. The expression of serum miR-150-5p is downregulated in patients with CCA, demonstrating a 91.43% sensitivity and 80% specificity for diagnosis. When combined with CA19-9 expression, the sensitivity increases to 93.33% and specificity to 96.88% [129]. To discriminate dCCA from pancreatic ductal adenocarcinoma and improve diagnosis, a recent study revealed that miR-16 is downregulated and miR-877 is upregulated in patients with dCCA. The combination of the two miRNAs—miR-16 and miR-877—has shown an AUC = 0.90, 79% sensitivity, and 90% specificity for diagnosis, and an AUC = 0.88, 71% sensitivity, and 90% specificity for discriminating dCCA from pancreatic ductal adenocarcinoma [138].

### 4.4. miRNAs in CCA Therapy Resistance 

For certain patients with advanced-stage CCA, for whom surgical resection or liver transplantation is not feasible, a combination of gemcitabine and cisplatin (GemCis) is used as a first-line systemic therapy irrespective of the CCA subtype [2]. Furthermore, a combination of 5-fluorouracil, leucovorin, oxaliplatin, and irinotecan (FOLFIRINOX) [139] or a combination of gemcitabine, cisplatin, and nab-paclitaxel [140] are associated with improved patient survival. In recent years, an increasing number of studies have focused on genetic pathophysiology, and carcinogenic and mutant genes have been identified in many malignancies, including CCA, which in turn promote the development of targeted therapy for CCA. However, drug resistance remains a key issue during such treatment. Numerous studies have reported the role of miRNAs in CCA therapy resistance. For instance, miR-210 sustains HIF-1α activity by targeting *HIF-3α*, which reduces the sensitivity to gemcitabine in CCA cells [141]. miR-130a-3p increases gemcitabine resistance by targeting *PPARG* [142]. Overexpression of miR-199a-3p enhances cisplatin sensitivity by inhibiting the mTOR signaling pathway in CCA cells [143]. miR-106b overexpression increases 5-fluorouracil sensitivity by targeting *ZBTB7A*, and miR-106b downregulation is related to poor prognosis in patients with CCA [144] (Table 4). 

### 4.5. miRNA-Based Therapies

As previously mentioned, miRNAs play a key role as oncogenic or suppressor miRNAs in the development and progression of CCA, and they act as regulators of drug resistance. miRNA-based therapy is a novel targeted therapy for cancers that is based on the concept of overexpressing suppressor miRNAs or decreasing the expression of oncogenic miRNAs using miRNA sponges. Although numerous in vitro and xenograft model-based studies [82,84,91] that have used miRNA mimics, inhibitors, and plasmids have indicated the functions of miRNAs, miRNA-based therapy has not yet achieved clinical translation. Because of the heterogeneity of the tumor cells and the complexity of miRNA functions, the same miRNA can have opposite functions in different malignancies. Furthermore, one miRNA can target different genes to regulate the protein expression. Additionally, the complexity of the in vivo environment can also affect the therapy. Finally, determining the approach and accuracy of drug delivery, evaluating efficacious doses, and predicting off-target effects are aspects that need to be considered. Currently, these act as obstacles that prevent the use of miRNA-based therapy in clinical applications [145,146]. 

## 5. Conclusions

Studying the biological functions of miRNAs, especially their roles in malignancies, is a growing field of research. miRNAs have been reported to play key roles in tumorigenesis, cell proliferation, apoptosis, metastasis, angiogenesis, EMT, and autophagy. In this review, we summarized the functional roles and related target genes of oncogenic and suppressor miRNAs implicated in the development and progression of CCA. Dysregulated expression of miRNAs in CCA has been utilized as potential biomarker for clinical diagnosis and prognosis prediction. Furthermore, miRNAs and their target genes contribute toward the development of targeted therapy and determination of drug resistance mechanisms. Although accumulating evidence has demonstrated that miRNAs may be potential biological targets for CCA treatment in preclinical studies, they are not yet suitable for clinical practice because of tumor cell heterogeneity as well as the complexity of the in vivo environment and miRNA functions. Meanwhile, determining drug delivery approaches, evaluating efficacious doses, and predicting off-target effects remain obstacles that prevent the clinical application of miRNA-based therapy. Further research and analyses of miRNAs will provide more evidence and novel insights into the pathogenesis of CCA and will prove to be useful for the diagnosis, therapy, and prognosis prediction in patients with CCA.

## Figures and Tables

**Table 1:** microRNAs (miRNAs) associated with cholangiocarcinoma risk factors.

Risk Factor	Related microRNA	Expression	Functions	References
**Lithiasis**				
Cholecystolithiasis	miR-210	upregulated	Targets *ATP11A* to regulate the ABC transporter pathway	[43]
Hepatolithiasis	miR-130b	downregulated	miR-130b-Sp1-MUC5AC signaling pathway	[44]
Choledocholithiasis	unknown	unknown	unknown	
**Cholestatic liver diseases**				
PSC	miR-200b	upregulated	Promotes biliary hyperplasia and liver fibrosis	[46]
FPLD	unknown	unknown	unknown	
**Parasitic infection**				
	miR-21 miR-96 miR-351 miR-146a/b miR-27b	unknown	Promotes fibrosis	[47,48]
**Cirrhosis**				
	miR-378	upregulated	NF-κB-TNFα axis	[49]
	miR-30a	downregulated	Targets Beclin1-mediated autophagy	[50]
	miR-214-3p	upregulated	Decreases Sufu and promotes HSC activation	[51]
**Infections**				
HBV	miR-192-3p	downregulated	Inhibits autophagy and suppresses HBV replication	[54]
	miR-224, miR-1231	unknown	Suppresses HBV replication	[55,56]
	miR-99 family	unknown	Promotes HBV replication	[57]
HCV	miR-215	unknown	Promotes HCV replication via targeting *TRIM22*	[59]
	miR-21-5p	upregulated	Promotes HCV replication	[60]
	miR-199a	unknown	Suppresses HCV replication	[62]
	miR-130a	unknown	Suppresses HCV replication	[63]
**Inflammatory disorders**				
IBD	miR-301a	upregulated	Promotes mucosal inflammation	[65]
	miR-31	upregulated	Reduces inflammatory response	[66]
Chronic pancreatitis	miR-15/16	downregulated	Alleviates apoptosis and fibrosis	[67]
**Metabolic abnormalities**				
Diabetes	miR-10b-5p	unknown	Regulates diabetes by KLF11-KIT pathway	[69]
	miR-690	unknown	Improves insulin sensitivity	[70]
NAFLD	miR-132	upregulated	Alters serum and hepatic lipid profiles	[72]
**Lifestyle**				
Alcohol	miR-148a	downregulated	Regulates hepatocyte apoptosis	[73]
Tobacco	miR-25-3p	unknown	Promotes the development and progression of cancers	[75,76]

Abbreviations: PSC, primary sclerosing cholangitis; FPLD, fibropolycystic liver diseases; HBV, hepatitis B virus; HCV, hepatitis C virus; IBD, inflammatory bowel disease; NAFLD, non-alcoholic fatty liver disease; HSC, hepatic stellate cell.

**Table 2:** Reported miRNAs acting as oncogenic or suppressor miRNAs in cholangiocarcinoma.

miRNA	Target Gene	Mechanism	References
**Oncogenic miRNA**			
miR-383	Interferon regulatory factor 1 (*IRF1*)	Proliferation, migration, and invasion	[77]
miR-221	Phosphatase and tensin homolog (*PTEN*)	Epithelial–mesenchymal transition (EMT)	[78]
miR-31	RAS p21 GTPase activating protein 1 (*RASA1*)	Proliferation and apoptosis	[79]
miR-361-5p	TNF receptor-associated factor 3 (*TRAF3*)	Apoptosis	[80]
miR-30a-5p	Suppressor of cytokine signaling 3 (*SOCS3*)	Proliferation and apoptosis	[81]
miR-21	15-hydroxyprostaglandin dehydrogenase (*15-PGDH*/*HPGD*)	Cell growth	[82]
miR-18a-5p	Fructose-bisphosphatase 1 (*FBP1*)	Proliferation, invasion, and apoptosis	[83]
miR-421	Farnesoid X receptor (*FXR*)	Proliferation and migration	[84]
miR-191	Ten-eleven translocation 1 (*TET1*)	Proliferation, invasion, and migration	[85]
miR-181c	N-myc downstream-regulated gene 2 (*NDRG2*)	Proliferation, chemoresistance, and metastasis	[86]
miR-193-3p	Transforming growth factor-β receptor type 3 (*TGFBR3*)	Proliferation, migration, and invasion	[87]
**Suppressor miRNA**			
miR-876	B-cell lymphoma-extra large (*BCL-XL*)	Proliferation and apoptosis	[88]
miR-451a	Activating transcription factor 2 (*ATF2*), ST8 alpha-N-acetyl-neuraminide alpha-2,8-sialyltransferase 4 (*ST8SIA4*)	Migration, invasion, and proliferation	[89,90]
miR-34a	Notch receptor 1 (*NOTCH1*), Notch receptor 2 (*NOTCH2*), Jagged canonical Notch ligand 1 (*JAG1*)	Proliferation	[91]
miR-186	Twist family BHLH transcription factor 1 (*TWIST1*)	Proliferation, migration, invasion, and EMT	[92]
miR-15a	Plasminogen activator inhibitor type-2 (*PAI-2*)	Migration	[93]
miR-612	*BCL-2*	Proliferation, migration, and invasion	[94]
miR-424-5p	AMPK-related protein kinase 5 (*ARK5*)	Metastasis, invasion, migration, and EMT	[95]
miR-124-3p	Ubiquitin-like, containing PHD and RING finger domains 1 (*UHRF1*)	Proliferation and cell cycle arrest	[96]
miR-551b-3p	Cyclin D1 (*CCND1*)	Proliferation and apoptosis	[97]
miR-186-5p	Microrchidia family CW-type zinc finger 2 (*MORC2*)	Cell growth and metastasis	[98]
miR-137	Wnt family member 2B (*WNT2B*)	Proliferation, migration, and invasion	[99]
miR-490-3p	*AKIRIN2*	Proliferation, migration, invasion, and angiogenesis	[100]
miR-329	Laminin subunit beta 3 (*LAMB3*)	Proliferation, migration, and invasion	[101]
miR-122-5p	Aldolase, Fructose-Bisphosphate A (*ALDOA*)	Proliferation, invasion, apoptosis, and EMT	[102,103]
miR-410	X-linked inhibitor of apoptosis protein (*XIAP*)	Apoptosis	[104]
miR-22	Histone deacetylase 6 (*HDAC6*)	Proliferation and migration	[105]
miR-433	Histone deacetylase 6 (*HDAC6*)	Proliferation and migration	[105]
miR-144	Platelet-activating factor acetylhydrolase isoform 1b (*LIS1*), ST8SIA4	Proliferation and invasion	[90,106]
miR-590-3p	Sphingosine-1-phosphate receptor 1 (*SIP1*)	Invasion, migration, and EMT	[107]
miR-101	Vascular endothelial growth factor (*VEGF*), Cyclooxygenase-2 (*COX-2*), E2F transcription factor 8 (*E2F8*)	Angiogenesis and proliferation	[108,109]
miR-26b-5p	S100 calcium-binding protein A7 (*S100A7*)	Proliferation, migration, and invasion	[110]
miR-1182	*NUAK1* (also known as *ARK5*)	Invasion, migration, and autophagy	[111]
let-7a	*NUAK1*	Invasion, migration, and autophagy	[111]
miR-874	Cyclin E1 (*CCNE1*)	Invasion and EMT	[112]
miR-885-5p	Insulin like growth factor 2 mRNA binding protein 1 (*IGF2BP1*), Polypeptide N-acetylgalactosaminyltransferase 3 (*GALNT3*)	Proliferation and metastasis	[113]
miR-622	*C-MYC*	Proliferation, migration, and invasion	[114]
miR-320	Neuropilin-1 (*NRP-1*)	Proliferation, invasion, and angiogenesis	[115]
miR-200b/c	SUZ12 Polycomb repressive complex 2 subunit (*SUZ12*)/Rho associated coiled-coil containing protein kinase 2 (*ROCK2*)	Tumorigenesis and metastasis	[116]

**Table 3:** The expression levels of potential miRNA-based biomarkers in cholangiocarcinoma.

miRNA	Expression	Detectable Location	Tumor Type (Background)	Biomarker Category	References
miR-21, miR-221	Upregulated	Plasma	Hepatolithiasis-CCA	Diagnosis/Prognosis	[45,121]
miR-26a	Upregulated	Serum	CCA	Diagnosis/Prognosis	[128]
miR-150-5p	Downregulated	Serum	CCA	Diagnosis	[129]
miR-29a	Upregulated	Tissue	CCA	Prognosis	[130]
miR-192	Upregulated	Serum	CCA	Prognosis	[131]
miR-151-3p	Upregulated	Tissue	Resected CCA	Prognosis	[132]
miR-126	Downregulated	Tissue	Resected CCA	Prognosis	[132]
miR-106a	Downregulated	Serum	CCA	Prognosis	[133]
miR-146a	Upregulated	Tissue	iCCA	Prognosis	[134]
miR-31	Upregulated	Tissue	CCA	Prognosis	[135]
miR-203	Downregulated	Tissue	CCA	Prognosis	[136]
miR-191	Upregulated	Tissue	iCCA	Prognosis	[85]
miR-195	Downregulated	Serum	CCA	Prognosis	[137]
miR-16	Downregulated	Plasma	dCCA	Diagnosis	[138]
miR-877	Upregulated	Plasma	dCCA	Diagnosis	[138]

Abbreviations: CCA, cholangiocarcinoma; iCCA, intrahepatic cholangiocarcinoma; dCCA distal cholangiocarcinoma.

**Table 4:** miRNAs in cholangiocarcinoma therapy resistance.

Drug	miRNA	Resistance	Target	Reference
Gemcitabine	miR-210	increase	*HIF-3α*	[141]
	miR-130a-3p	increase	*PPARG*	[142]
Cisplatin	miR-199a-3p	decrease	mTOR signaling pathway	[143]
5-fluorouracil	miR-106b	decrease	*ZBTB7A*	[144]

## References

[1] Banales J.M., Marin J.J.G., Lamarca A., Rodrigues P.M., Khan S.A., Roberts L.R., Cardinale V., Carpino G., Andersen J.B., Braconi C. (2020). Cholangiocarcinoma 2020: The next horizon in mechanisms and management. Nat. Rev. Gastroenterol. Hepatol..

[2] Rizvi S., Khan S.A., Hallemeier C.L., Kelley R.K., Gores G.J. (2018). Cholangiocarcinoma—Evolving concepts and therapeutic strategies. Nat. Rev. Clin. Oncol..

[3] Sung H., Ferlay J., Siegel R.L., Laversanne M., Soerjomataram I., Jemal A., Bray F. (2021). Global cancer statistics 2020: GLOBOCAN estimates of incidence and mortality worldwide for 36 cancers in 185 countries. CA Cancer J. Clin..

[4] Moeini A., Sia D., Zhang Z., Campreciós G., Stueck A., Dong H., Montal R., Torrens L., Martinez-Quetglas I., Fiel M.I. (2017). Mixed hepatocellular cholangiocarcinoma tumors: Cholangiolocellular carcinoma is a distinct molecular entity. J. Hepatol..

[5] Esnaola N.F., Meyer J.E., Karachristos A., Maranki J.L., Camp E.R., Denlinger C.S. (2016). Evaluation and management of intrahepatic and extrahepatic cholangiocarcinoma. Cancer.

[6] Spolverato G., Kim Y., Alexandrescu S., Marques H.P., Lamelas J., Aldrighetti L., Gamblin T.C., Maithel S.K., Pulitano C., Bauer T.W. (2016). Management and Outcomes of Patients with Recurrent Intrahepatic Cholangiocarcinoma Following Previous Curative-Intent Surgical Resection. Ann. Surg. Oncol..

[7] Lee Y.S., Dutta A. (2009). MicroRNAs in Cancer. Annu. Rev. Pathol. Mech. Dis..

[8] Di Leva G., Garofalo M., Croce C.M. (2014). MicroRNAs in Cancer. Annu. Rev. Pathol..

[9] Syeda Z.A., Langden S.S.S., Munkhzul C., Lee M., Song S.J. (2020). Regulatory Mechanism of MicroRNA Expression in Cancer. Int. J. Mol. Sci..

[10] De Sousa M.C., Gjorgjieva M., Dolicka D., Sobolewski C., Foti M. (2019). Deciphering miRNAs’ Action through miRNA Editing. Int. J. Mol. Sci..

[11] Yu J., Wang F., Yang G.-H., Wang F.-L., Ma Y.-N., Du Z.-W., Zhang J.-W. (2006). Human microRNA clusters: Genomic organization and expression profile in leukemia cell lines. Biochem. Biophys. Res. Commun..

[12] Cozar J., Robles-Fernandez I., Rodriguez-Martinez A., Puche-Sanz I., Vazquez-Alonso F., Lorente J., Martinez-Gonzalez L., Alvarez-Cubero M. (2019). The role of miRNAs as biomarkers in prostate cancer. Mutat. Res. Mutat. Res..

[13] Bertuccio P., Malvezzi M., Carioli G., Hashim D., Boffetta P., El-Serag H.B., La Vecchia C., Negri E. (2019). Global trends in mortality from intrahepatic and extrahepatic cholangiocarcinoma. J. Hepatol..

[14] Yao K.J., Jabbour S., Parekh N., Lin Y., Moss R.A. (2016). Increasing mortality in the United States from cholangiocarcinoma: An analysis of the National Center for Health Statistics Database. BMC Gastroenterol..

[15] Blechacz B. (2017). Cholangiocarcinoma: Current Knowledge and New Developments. Gut Liver.

[16] Witjes C.D.M., Karim-Kos H.E., Visser O., De Vries E., Ijzermans J.N.M., De Man R.A., Coebergh J.W.W., Verhoef C. (2012). Intrahepatic cholangiocarcinoma in a low endemic area: Rising incidence and improved survival. HPB.

[17] Tyson G.L., Ilyas J.A., Duan Z., Green L.K., Younes M., El-Serag H.B., Davila J.A. (2014). Secular Trends in the Incidence of Cholangiocarcinoma in the USA and the Impact of Misclassification. Dig. Dis. Sci..

[18] Kaneko R., Sato Y., Kobayashi Y. (2018). Cholangiocarcinoma Prognosis Varies over Time Depending on Tumor Site and Pathology. J. Gastrointest. Liver Dis..

[19] Cai W.-K., Sima H., Chen B.-D., Yang G.-S. (2011). Risk factors for hilar cholangiocarcinoma: A case-control study in China. World J. Gastroenterol..

[20] Arbelaiz A., Azkargorta M., Krawczyk M., Santos-Laso A., Lapitz A., Perugorria M.J., Erice O., Gonzalez E., Jimenez-Agüero R., La Casta A. (2017). Serum extracellular vesicles contain protein biomarkers for primary sclerosing cholangitis and cholangiocarcinoma. Hepatology.

[21] Fabris L., Fiorotto R., Spirli C., Cadamuro M., Mariotti V., Perugorria M.J., Banales J.M., Strazzabosco M. (2019). Pathobiology of inherited biliary diseases: A roadmap to understand acquired liver diseases. Nat. Rev. Gastroenterol. Hepatol..

[22] Prueksapanich P., Piyachaturawat P., Aumpansub P., Ridtitid W., Chaiteerakij R., Rerknimitr R. (2018). Liver Fluke-Associated Biliary Tract Cancer. Gut Liver.

[23] Shaib Y.H., El-Serag H.B., Davila J.A., Morgan R., McGlynn K.A. (2005). Risk factors of intrahepatic cholangiocarcinoma in the United States: A case-control study. Gastroenterology.

[24] Tan J.-H., Zhou W.-Y., Zhou L., Cao R.-C., Zhang G.-W. (2020). Viral hepatitis B and C infections increase the risks of intrahepatic and extrahepatic cholangiocarcinoma: Evidence from a systematic review and meta-analysis. Turk. J. Gastroenterol..

[25] Petrick J.L., Yang B., Altekruse S.F., Van Dyke A.L., Koshiol J., Graubard B.I., McGlynn K.A. (2017). Risk factors for intrahepatic and extrahepatic cholangiocarcinoma in the United States: A population-based study in SEER-Medicare. PLoS ONE.

[26] Wongjarupong N., Assavapongpaiboon B., Susantitaphong P., Cheungpasitporn W., Treeprasertsuk S., Rerknimitr R., Chaiteerakij R. (2017). Non-alcoholic fatty liver disease as a risk factor for cholangiocarcinoma: A systematic review and meta-analysis. BMC Gastroenterol..

[27] Petrick J.L., Campbell P.T., Koshiol J., Thistle J.E., Andreotti G., Beane-Freeman L.E., Buring J.E., Chan A.T., Chong D.Q., Doody M.M. (2018). Tobacco, alcohol use and risk of hepatocellular carcinoma and intrahepatic cholangiocarcinoma: The Liver Cancer Pooling Project. Br. J. Cancer.

[28] Labib P.L., Goodchild G., Pereira S.P. (2019). Molecular Pathogenesis of Cholangiocarcinoma. BMC Cancer.

[29] Suzuki Y., Mori T., Abe N., Sugiyama M., Atomi Y. (2012). Predictive factors for cholangiocarcinoma associated with hepatolithiasis determined on the basis of Japanese Multicenter study. Hepatol. Res..

[30] Karlsen T.H., Folseraas T., Thorburn D., Vesterhus M. (2017). Primary sclerosing cholangitis—A comprehensive review. J. Hepatol..

[31] Zhang H., Yang T., Wu M., Shen F. (2016). Intrahepatic cholangiocarcinoma: Epidemiology, risk factors, diagnosis and surgical management. Cancer Lett..

[32] Goeppert B., Folseraas T., Roessler S., Kloor M., Volckmar A., Endris V., Buchhalter I., Stenzinger A., Grzyb K., Grimsrud M.M. (2020). Genomic Characterization of Cholangiocarcinoma in Primary Sclerosing Cholangitis Reveals Therapeutic Opportunities. Hepatology.

[33] Trivedi P.J., Crothers H., Mytton J., Bosch S., Iqbal T., Ferguson J., Hirschfield G.M. (2020). Effects of Primary Sclerosing Cholangitis on Risks of Cancer and Death in People With Inflammatory Bowel Disease, Based on Sex, Race, and Age. Gastroenterology.

[34] Sripa B., Kaewkes S., Sithithaworn P., Mairiang E., Laha T., Smout M., Pairojkul C., Bhudhisawasdi V., Tesana S., Thinkamrop B. (2007). Liver Fluke Induces Cholangiocarcinoma. PLoS Med..

[35] Alsaleh M., Leftley Z., Barbera T.A., Sithithaworn P., Khuntikeo N., Loilome W., Yongvanit P., Cox I.J., Chamodol N., Syms R.R. (2018). Cholangiocarcinoma: A guide for the nonspecialist. Int. J. Gen. Med..

[36] Na B.-K., Pak J.H., Hong S.-J. (2020). Clonorchis sinensis and clonorchiasis. Acta Trop..

[37] Kurokawa T., Sato T., Andoh H., Yasui O. (2004). Cholangiocarcinoma coincident with schistosomiasis japonica. J. Gastroenterol..

[38] Easterbrook P.J., Roberts T., Sands A., Peeling R. (2017). Diagnosis of viral hepatitis. Curr. Opin. HIV AIDS.

[39] Li M., Li J., Li P., Li H., Su T., Zhu R., Gong J. (2012). Hepatitis B virus infection increases the risk of cholangiocarcinoma: A meta-analysis and systematic review. J. Gastroenterol. Hepatol..

[40] Zhang H., Zhu B., Zhang H., Liang J., Zeng W. (2016). HBV Infection Status and the Risk of Cholangiocarcinoma in Asia: A Meta-Analysis. BioMed Res. Int..

[41] Palmer W.C., Patel T. (2012). Are common factors involved in the pathogenesis of primary liver cancers? A meta-analysis of risk factors for intrahepatic cholangiocarcinoma. J. Hepatol..

[42] Welzel T.M., Graubard B.I., El–Serag H.B., Shaib Y.H., Hsing A.W., Davila J.A., McGlynn K.A. (2007). Risk Factors for Intrahepatic and Extrahepatic Cholangiocarcinoma in the United States: A Population-Based Case-Control Study. Clin. Gastroenterol. Hepatol..

[43] Yang B., Liu B., Bi P., Wu T., Wang Q., Zhang J. (2015). An integrated analysis of differential miRNA and mRNA expressions in human gallstones. Mol. BioSyst..

[44] Wu X., Yao C., Kong J., Tian Y., Fan Y., Zhang Z., Han J., Wu S. (2021). Molecular mechanism underlying miR-130b-Sp1 transcriptional regulation in LPS-induced upregulation of MUC5AC in the bile duct epithelium. Mol. Med. Rep..

[45] Jiang W., Deng X., Zhu T., Wei Y., Lei Z., Guo M., Yang J. (2020). Identification of Cholangiocarcinoma Associated with Hepatolithiasis via the Combination of miRNA and Ultrasound. Cancer Manag. Res..

[46] Wu N., Meng F., Zhou T., Han Y., Kennedy L., Venter J., Francis H., DeMorrow S., Onori P., Invernizzi P. (2017). Prolonged darkness reduces liver fibrosis in a mouse model of primary sclerosing cholangitis by miR-200b down-regulation. FASEB J..

[47] Chen Q., Zhang J., Zheng T., Chen H., Nie H., Zheng B., Gong Q. (2019). The role of microRNAs in the pathogenesis, grading and treatment of hepatic fibrosis in schistosomiasis. Parasites Vectors.

[48] Zhu D., Lyu L., Shen P., Wang J., Chen J., Sun X., Chen L., Zhang L., Zhou Q., Duan Y. (2018). rSjP40 protein promotes PPARγ expression in LX-2 cells through microRNA-27b. FASEB J..

[49] Zhang T., Hu J., Wang X., Zhao X., Li Z., Niu J., Steer C.J., Zheng G., Song G. (2019). MicroRNA-378 promotes hepatic inflammation and fibrosis via modulation of the NF-κB-TNFα pathway. J. Hepatol..

[50] Chen J., Yu Y., Li S., Liu Y., Zhou S., Cao S., Yin J., Li G. (2017). MicroRNA-30a ameliorates hepatic fibrosis by inhibiting Beclin1-mediated autophagy. J. Cell. Mol. Med..

[51] Ma L., Yang X., Wei R., Ye T., Zhou J., Wen M., Men R., Li P., Dong B., Liu L. (2018). MicroRNA-214 promotes hepatic stellate cell activation and liver fibrosis by suppressing Sufu expression. Cell Death Dis..

[52] Liang T.J. (2009). Hepatitis B: The virus and disease. Hepatology.

[53] Wang G., Dong F., Xu Z., Sharma S., Hu X., Chen D., Zhang L., Zhang J., Dong Q. (2017). MicroRNA profile in HBV-induced infection and hepatocellular carcinoma. BMC Cancer.

[54] Wang J., Chen J., Liu Y., Zeng X., Wei M., Wu S., Xiong Q., Song F., Yuan X., Xiao Y. (2019). Hepatitis B Virus Induces Autophagy to Promote its Replication by the Axis of miR-192-3p-XIAP Through NF kappa B Signaling. Hepatology.

[55] Gao K., Liu F., Guo H., Li J., Zhang Y., Mo Z. (2018). miR-224 suppresses HBV replication posttranscriptionally through inhibiting SIRT1-mediated autophagy. Int. J. Clin. Exp. Pathol..

[56] Kohno T., Tsuge M., Murakami E., Hiraga N., Abe H., Miki D., Imamura M., Ochi H., Hayes C.N., Chayama K. (2014). Human microRNA hsa-miR-1231 suppresses hepatitis B virus replication by targeting core mRNA. J. Viral Hepat..

[57] Lin Y., Deng W., Pang J., Kemper T., Hu J., Yin J., Zhang J., Lu M. (2017). The microRNA-99 family modulates hepatitis B virus replication by promoting IGF-1R/PI3K/Akt/mTOR/ULK1 signaling-induced autophagy. Cell. Microbiol..

[58] Roudot-Thoraval F. (2021). Epidemiology of hepatitis C virus infection. Clin. Res. Hepatol. Gastroenterol..

[59] Tian H., He Z. (2018). miR-215 Enhances HCV Replication by Targeting TRIM22 and Inactivating NF-κB Signaling. Yonsei Med. J..

[60] Clément S., Sobolewski C., Gomes D., Rojas A., Goossens N., Conzelmann S., Calo N., Negro F., Foti M. (2019). Activation of the oncogenic miR-21-5p promotes HCV replication and steatosis induced by the viral core 3a protein. Liver Int..

[61] Kunden R.D., Khan J.Q., Ghezelbash S., Wilson J.A. (2020). The Role of the Liver-Specific microRNA, miRNA-122 in the HCV Replication Cycle. Int. J. Mol. Sci..

[62] Murakami Y., Aly H.H., Tajima A., Inoue I., Shimotohno K. (2009). Regulation of the hepatitis C virus genome replication by miR-199a. J. Hepatol..

[63] Duan X., Liu X., Li W., Holmes J.A., Kruger A.J., Yang C., Li Y., Xu M., Ye H., Li S. (2019). Microrna-130a Downregulates HCV Replication through an atg5-Dependent Autophagy Pathway. Cells.

[64] Coussens L.M., Werb Z. (2002). Inflammation and cancer. Nature.

[65] He C., Shi Y., Wu R., Sun M., Fang L., Wu W., Liu C., Tang M., Li Z., Wang P. (2016). miR-301a promotes intestinal mucosal inflammation through induction of IL-17A and TNF-α in IBD. Gut.

[66] Tian Y., Xu J., Li Y., Zhao R., Du S., Lv C., Wu W., Liu R., Sheng X., Song Y. (2019). MicroRNA-31 Reduces Inflammatory Signaling and Promotes Regeneration in Colon Epithelium, and Delivery of Mimics in Microspheres Reduces Colitis in Mice. Gastroenterology.

[67] Ji T., Feng W., Zhang X., Zang K., Zhu X., Shang F. (2020). HDAC inhibitors promote pancreatic stellate cell apoptosis and relieve pancreatic fibrosis by upregulating miR-15/16 in chronic pancreatitis. Hum. Cell.

[68] Feng J., Xing W., Xie L. (2016). Regulatory Roles of MicroRNAs in Diabetes. Int. J. Mol. Sci..

[69] Singh R., Ha S.E., Wei L., Jin B., Zogg H., Poudrier S.M., Jorgensen B.G., Park C., Ronkon C.F., Bartlett A. (2021). miR-10b-5p Rescues Diabetes and Gastrointestinal Dysmotility. Gastroenterology.

[70] Ying W., Gao H., Dos Reis F.C.G., Bandyopadhyay G., Ofrecio J.M., Luo Z., Ji Y., Jin Z., Ly C., Olefsky J.M. (2021). MiR-690, an exosomal-derived miRNA from M2-polarized macrophages, improves insulin sensitivity in obese mice. Cell Metab..

[71] Su Q., Kumar V., Sud N., Mahato R.I. (2018). MicroRNAs in the pathogenesis and treatment of progressive liver injury in NAFLD and liver fibrosis. Adv. Drug Deliv. Rev..

[72] Hanin G., Yayon N., Tzur Y., Haviv R., Bennett E.R., Udi S., Krishnamoorthy Y.R., Kotsiliti E., Zangen R., Efron B. (2018). miRNA-132 induces hepatic steatosis and hyperlipidaemia by synergistic multitarget suppression. Gut.

[73] Heo M.J., Kim T.H., You J.S., Blaya D., Sancho-Bru P., Kim S.G. (2019). Alcohol dysregulates miR-148a in hepatocytes through FoxO1, facilitating pyroptosis via TXNIP overexpression. Gut.

[74] Bala S., Csak T., Saha B., Zatsiorsky J., Kodys K., Catalano D., Satishchandran A., Szabo G. (2016). The pro-inflammatory effects of miR-155 promote liver fibrosis and alcohol-induced steatohepatitis. J. Hepatol..

[75] Zhang J., Bai R., Li M., Ye H., Wu C., Wang C., Li S., Tan L., Mai D., Li G. (2019). Excessive miR-25-3p maturation via N6-methyladenosine stimulated by cigarette smoke promotes pancreatic cancer progression. Nat. Commun..

[76] Zeng Z., Li Y., Pan Y., Lan X., Song F., Sun J., Zhou K., Liu X., Ren X., Wang F. (2018). Cancer-derived exosomal miR-25-3p promotes pre-metastatic niche formation by inducing vascular permeability and angiogenesis. Nat. Commun..

[77] Wan P., Chi X., Du Q., Luo J., Cui X., Dong K., Bing Y., Heres C., Geller D.A. (2018). miR-383 promotes cholangiocarcinoma cell proliferation, migration, and invasion through targeting IRF. J. Cell. Biochem..

[78] Li J., Yao L., Li G., Ma D., Sun C., Gao S., Zhang P., Gao F. (2015). miR-221 Promotes Epithelial-Mesenchymal Transition through Targeting PTEN and Forms a Positive Feedback Loop with β-catenin/c-Jun Signaling Pathway in Extra-Hepatic Cholangiocarcinoma. PLoS ONE.

[79] Hu C., Huang F., Deng G., Nie W., Huang W., Zeng X. (2013). miR-31 promotes oncogenesis in intrahepatic cholangiocarcinoma cells via the direct suppression of RASA1. Exp. Ther. Med..

[80] Lu W.-X. (2019). Long non-coding RNA MEG3 represses cholangiocarcinoma by regulating miR-361-5p/TRAF3 axis. Eur. Rev. Med. Pharmacol. Sci..

[81] Zhang J.W., Wang X., Li G.C., Wang D., Han S., Zhang Y.D., Luo C.H., Wang H.W., Jiang W.J., Li C.X. (2020). MiR-30a-5p promotes cholangiocarcinoma cell proliferation through targeting SOCS3. J. Cancer.

[82] Lu L., Byrnes K., Han C., Wang Y., Wu T. (2014). miR-21 Targets 15-PGDH and Promotes Cholangiocarcinoma Growth. Mol. Cancer Res..

[83] Zhao W., Zhao J., Guo X., Feng Y., Zhang B., Tian L. (2021). LncRNA MT1JP plays a protective role in intrahepatic cholangiocarcinoma by regulating miR-18a-5p/FBP1 axis. BMC Cancer.

[84] Zhong X.-Y., Yu J.-H., Zhang W.-G., Wang Z.-D., Dong Q., Tai S., Cui Y.-F., Li H. (2012). MicroRNA-421 functions as an oncogenic miRNA in biliary tract cancer through down-regulating farnesoid X receptor expression. Gene.

[85] Li H., Zhou Z.-Q., Yang Z.-R., Tong D.-N., Guan J., Shi B.-J., Nie J., Ding X.-T., Li B., Zhou G.-W. (2017). MicroRNA-191 acts as a tumor promoter by modulating the TET1-p53 pathway in intrahepatic cholangiocarcinoma. Hepatology.

[86] Wang J., Xie C., Pan S., Liang Y., Han J., Lan Y., Sun J., Li K., Sun B., Yang G. (2016). N-myc downstream-regulated gene 2 inhibits human cholangiocarcinoma progression and is regulated by leukemia inhibitory factor/MicroRNA-181c negative feedback pathway. Hepatology.

[87] Han Y., Yin J., Cong J. (2018). Downregulation of microRNA-193-3p inhibits the progression of intrahepatic cholangiocarcinoma cells by upregulating TGFBR3. Exp. Ther. Med..

[88] Ursu S., Majid S., Garger C., De Semir D., Bezrookove V., Desprez P.-Y., McAllister S., Soroceanu L., Nosrati M., Yimam K. (2019). Novel tumor suppressor role of miRNA-876 in cholangiocarcinoma. Oncogenesis.

[89] Loeffler M.A., Hu J., Kirchner M., Wei X., Xiao Y., Albrecht T., De La Torre C., Sticht C., Banales J.M., Vogel M.N. (2020). miRNA profiling of biliary intraepithelial neoplasia reveals stepwise tumorigenesis in distal cholangiocarcinoma via the miR-451a/ATF2 axis. J. Pathol..

[90] Fu W., Yu G., Liang J., Fan P., Dong K., Zhang B., Chen X., Zhu H., Chu L. (2021). miR-144-5p and miR-451a Inhibit the Growth of Cholangiocarcinoma Cells Through Decreasing the Expression of ST8SIA4. Front. Oncol..

[91] Kwon H., Song K., Han C., Zhang J., Lu L., Chen W., Wu T. (2017). Epigenetic Silencing of miRNA-34a in Human Cholangiocarcinoma via EZH2 and DNA Methylation: Impact on Regulation of Notch Pathway. Am. J. Pathol..

[92] Zhang M., Shi B., Zhang K. (2019). miR-186 Suppresses the Progression of Cholangiocarcinoma Cells through Inhibition of Twist1. Oncol. Res..

[93] Utaijaratrasmi P., Vaeteewoottacharn K., Tsunematsu T., Jamjantra P., Wongkham S., Pairojkul C., Khuntikeo N., Ishimaru N., Sirivatanauksorn Y., Pongpaibul A. (2018). The microRNA-15a-PAI-2 axis in cholangiocarcinoma-associated fibroblasts promotes migration of cancer cells. Mol. Cancer.

[94] Yu A., Zhao L., Kang Q., Li J., Chen K., Fu H. (2020). Transcription factor HIF1α promotes proliferation, migration, and invasion of cholangiocarcinoma via long noncoding RNA H19/microRNA-612/Bcl-2 axis. Transl. Res..

[95] Wu J., Yang B., Zhang Y., Feng X., He B., Xie H., Zhou L., Wu J., Zheng S. (2019). miR-424-5p represses the metastasis and invasion of intrahepatic cholangiocarcinoma by targeting ARK5. Int. J. Biol. Sci..

[96] Zhu M., Wei C., Lin J., Dong S., Gao D., Chen J., Zhao Y., Liu B. (2019). UHRF1 is regulated by miR-124-3p and promotes cell proliferation in intrahepatic cholangiocarcinoma. J. Cell. Physiol..

[97] Chang W., Wang Y., Li W., Shi L., Geng Z. (2019). Micro RNA-551b-3p inhibits tumour growth of human cholangiocarcinoma by targeting Cyclin D1. J. Cell. Mol. Med..

[98] Liao G., Liu X., Wu D., Duan F., Xie X., Wen S., Li Y., Li S. (2019). MORC2 promotes cell growth and metastasis in human cholangiocarcinoma and is negatively regulated by miR-186-5p. Aging.

[99] Chen T., Lei S., Zeng Z., Pan S., Zhang J., Xue Y., Sun Y., Lan J., Xu S., Mao D. (2020). MicroRNA-137 suppresses the proliferation, migration and invasion of cholangiocarcinoma cells by targeting WNT2B. Int. J. Mol. Med..

[100] Leng K., Xu Y., Kang P., Qin W., Cai H., Wang H., Ji D., Jiang X., Li J., Li Z. (2019). Akirin2 is modulated by miR-490-3p and facilitates angiogenesis in cholangiocarcinoma through the IL-6/STAT3/VEGFA signaling pathway. Cell Death Dis..

[101] Liao C., Liu Y., Wu Y., Zhu S., Cai R., Zhou L., Yin X. (2019). microRNA-329 suppresses epithelial-to-mesenchymal transition and lymph node metastasis in bile duct cancer by inhibiting laminin subunit beta. J. Cell. Physiol..

[102] Peng C., Sun Z., Li O., Guo C., Yi W., Tan Z., Jiang B. (2019). Leptin stimulates the epithelial-mesenchymal transition and pro-angiogenic capability of cholangiocarcinoma cells through the miR-122/PKM2 axis. Int. J. Oncol..

[103] Xu Z., Liu G., Zhang M., Zhang Z., Jia Y., Peng L., Zhu Y., Hu J., Huang R., Sun X. (2018). miR-122-5p Inhibits the Proliferation, Invasion and Growth of Bile Duct Carcinoma Cells by Targeting ALDOA. Cell. Physiol. Biochem..

[104] Palumbo T., Poultsides G.A., Kouraklis G., Liakakos T., Drakaki A., Peros G., Hatziapostolou M., Iliopoulos D. (2016). A functional microRNA library screen reveals miR-410 as a novel anti-apoptotic regulator of cholangiocarcinoma. BMC Cancer.

[105] Mansini A.P., Pisarello M.J.L., Thelen K.M., Cruz-Reyes M., Peixoto E., Jin S., Howard B.N., Trussoni C.E., Gajdos G.B., LaRusso N.F. (2018). MicroRNA (miR)-433 and miR-22 dysregulations induce histone-deacetylase-6 overexpression and ciliary loss in cholangiocarcinoma. Hepatology.

[106] Yang R., Chen Y., Tang C., Li H., Wang B., Yan Q., Hu J., Zou S. (2014). MicroRNA-144 suppresses cholangiocarcinoma cell proliferation and invasion through targeting platelet activating factor acetylhydrolase isoform 1b. BMC Cancer.

[107] Zu C., Liu S., Cao W., Liu Z., Qiang H., Li Y., Cheng C., Ji L., Li J., Li J. (2017). MiR-590-3p suppresses epithelial-mesenchymal transition in intrahepatic cholangiocarcinoma by inhibiting SIP1 expression. Oncotarget.

[108] Zhang J., Han C., Zhu H., Song K., Wu T. (2013). miR-101 Inhibits Cholangiocarcinoma Angiogenesis through Targeting Vascular Endothelial Growth Factor (VEGF). Am. J. Pathol..

[109] Wang H., Wang L., Tang L., Luo J., Ji H., Zhang W., Zhou J., Li Q., Miao L. (2020). Long noncoding RNA SNHG6 promotes proliferation and angiogenesis of cholangiocarcinoma cells through sponging miR-101-3p and activation of E2F8. J. Cancer.

[110] Fan F., Lu J., Yu W., Zhang Y., Xu S., Pang L., Zhu B. (2018). MicroRNA-26b-5p regulates cell proliferation, invasion and metastasis in human intrahepatic cholangiocarcinoma by targeting S100A7. Oncol. Lett..

[111] Pan X., Wang G., Wang B. (2021). MicroRNA-1182 and let-7a exert synergistic inhibition on invasion, migration and autophagy of cholangiocarcinoma cells through down-regulation of NUAK1. Cancer Cell Int..

[112] Pan X., Wang G., Wang B. (2021). Ectopic expression of microRNA-874 represses epithelial mesenchymal transition through the NF-κB pathway via CCNE1 in cholangiocarcinoma. Cell. Signal..

[113] Lixin S., Wei S., Haibin S., Qingfu L., Tiemin P. (2020). miR-885-5p inhibits proliferation and metastasis by targeting IGF2BP1 and GALNT3 in human intrahepatic cholangiocarcinoma. Mol. Carcinog..

[114] Wu Y.-F., Li Z.-R., Cheng Z.-Q., Yin X.-M., Wu J.-S. (2017). Decrease of miR-622 expression promoted the proliferation, migration and invasion of cholangiocarcinoma cells by targeting regulation of c-Myc. Biomed. Pharmacother..

[115] Zhu H., Jiang X., Zhou X., Dong X., Xie K., Yang C., Jiang H., Sun X., Lu J. (2018). Neuropilin-1 regulated by miR-320 contributes to the growth and metastasis of cholangiocarcinoma cells. Liver Int..

[116] Peng F., Jiang J., Yu Y., Tian R., Guo X., Li X., Shen M., Xu M., Zhu F., Shi C. (2013). Direct targeting of SUZ12/ROCK2 by miR-200b/c inhibits cholangiocarcinoma tumourigenesis and metastasis. Br. J. Cancer.

[117] Wagenaar T.R., Zabludoff S., Ahn S.-M., Allerson C., Arlt H., Baffa R., Cao H., Davis S., Garcia-Echeverria C., Gaur R. (2015). Anti–miR-21 Suppresses Hepatocellular Carcinoma Growth via Broad Transcriptional Network Deregulation. Mol. Cancer Res..

[118] Najjary S., Mohammadzadeh R., Mokhtarzadeh A., Mohammadi A., Kojabad A.B., Baradaran B. (2020). Role of miR-21 as an authentic oncogene in mediating drug resistance in breast cancer. Gene.

[119] Selaru F.M., Olaru A.V., Kan T., David S., Cheng Y., Mori Y., Yang J., Paun B., Jin Z., Agarwal R. (2009). MicroRNA-21 is overexpressed in human cholangiocarcinoma and regulates programmed cell death 4 and tissue inhibitor of metalloproteinase 3. Hepatology.

[120] Wang L.-J., He C.-C., Sui X., Cai M.-J., Zhou C.-Y., Ma J.-L., Wu L., Wang H., Han S.-X., Zhu Q. (2015). MiR-21 promotes intrahepatic cholangiocarcinoma proliferation and growth in vitro and in vivo by targeting PTPN14 and PTEN. Oncotarget.

[121] Sun C., Zhu J., Wu B., Chen J., Zhu Z., Cai P., Guo W., Gu Z., Wang J., Huang S. (2018). Diagnostic and prognostic value of microRNAs in cholangiocarcinoma: A systematic review and meta-analysis. Cancer Manag. Res..

[122] Hu J., Wang Y.-N., Song D.-J., Tan J.-P., Cao Y., Fan J., Wang Z., Zhou J. (2021). A High-Accuracy Model Based on Plasma miRNAs Diagnoses Intrahepatic Cholangiocarcinoma: A Single Center with 1001 Samples. Diagnostics.

[123] Slabáková E., Culig Z., Remšík J., Souček K. (2017). Alternative mechanisms of miR-34a regulation in cancer. Cell Death Dis..

[124] Kong L., Wu Q., Zhao L., Ye J., Li N., Yang H. (2019). Upregulated lncRNA-UCA1 contributes to metastasis of bile duct carcinoma through regulation of miR-122/CLIC1and activation of the ERK/MAPK signaling pathway. Cell Cycle.

[125] Wang X., He Y., Mackowiak B., Gao B. (2021). MicroRNAs as regulators, biomarkers and therapeutic targets in liver diseases. Gut.

[126] Mishra N.K., Niu M., Southekal S., Bajpai P., Elkholy A., Manne U., Guda C. (2020). Identification of Prognostic Markers in Cholangiocarcinoma Using Altered DNA Methylation and Gene Expression Profiles. Front. Genet..

[127] Xu Y., Yao Y., Jiang X., Zhong X., Wang Z., Li C., Kang P., Leng K., Ji D., Li Z. (2018). SP1-induced upregulation of lncRNA SPRY4-IT1 exerts oncogenic properties by scaffolding EZH2/LSD1/DNMT1 and sponging miR-101-3p in cholangiocarcinoma. J. Exp. Clin. Cancer Res..

[128] Wang L.-J., Zhang K.-L., Zhang N., Ma X.-W., Yan S.-W., Cao D.-H., Shi S.-J. (2015). Serum miR-26a as a diagnostic and prognostic biomarker in cholangiocarcinoma. Oncotarget.

[129] Salem P.E.S., Ghazala R.A., El Gendi A.M., Emara D.M., Ahmed N.M. (2020). The association between circulating MicroRNA-150 level and cholangiocarcinoma. J. Clin. Lab. Anal..

[130] Deng Y., Chen Y. (2017). Increased Expression of miR-29a and Its Prognostic Significance in Patients with Cholangiocarcinoma. Oncol. Res. Treat..

[131] Silakit R., Loilome W., Yongvanit P., Chusorn P., Techasen A., Boonmars T., Khuntikeo N., Chamadol N., Pairojkul C., Namwat N. (2014). Circulating miR-192 in liver fluke-associated cholangiocarcinoma patients: A prospective prognostic indicator. J. Hepato Biliary Pancreat. Sci..

[132] McNally M.E., Collins A., Wojcik S.E., Liu J., Henry J.C., Jiang J., Schmittgen T., Bloomston M. (2013). Concomitant dysregulation of microRNAs miR-151-3p and miR-126 correlates with improved survival in resected cholangiocarcinoma. HPB.

[133] Cheng Q., Feng F., Zhu L., Zheng Y., Luo X., Liu C., Yi B., Jiang X. (2015). Circulating miR-106a is a Novel Prognostic and Lymph Node Metastasis Indicator for Cholangiocarcinoma. Sci. Rep..

[134] Zhang R.-X., Zheng Z., Li K., Wu X.-H., Zhu L. (2017). Both plasma and tumor tissue miR-146a high expression correlates with prolonged overall survival of surgical patients with intrahepatic cholangiocarcinoma. Medicine.

[135] Ishigami K., Nosho K., Kanno S., Mitsuhashi K., Igarashi H., Shitani M., Motoya M., Kimura Y., Hasegawa T., Kaneto H. (2018). MicroRNA-31 reflects IL-6 expression in cancer tissue and is related with poor prognosis in bile duct cancer. Carcinogenesis.

[136] Li J., Gao B., Huang Z., Duan T., Li D., Zhang S., Zhao Y., Liu L., Wang Q., Chen Z. (2015). Prognostic significance of microRNA-203 in cholangiocarcinoma. Int. J. Clin. Exp. Pathol..

[137] Chen Q., Wang C., Zhang H., Li Y., Cao Y., Zhang Y., Liu S., Li Z., Xin X., Han X. (2018). Expression levels of serum miRNA-195 in different types of patients with cholangiocarcinoma and its value to determine the prognosis thereof. Oncol. Lett..

[138] Meijer L.L., Puik J.R., Le Large T.Y., Heger M., Dijk F., Funel N., Wurdinger T., Garajová I., Van Grieken N.C., Van De Wiel M.A. (2019). Unravelling the Diagnostic Dilemma: A MicroRNA Panel of Circulating MiR-16 and MiR-877 as A Diagnostic Classifier for Distal Bile Duct Tumors. Cancers.

[139] Phelip J.-M., Edeline J., Blanc J.-F., Barbier E., Michel P., Bourgeois V., Neuzillet C., Malka D., Manfredi S., Desrame J. (2019). Modified FOLFIRINOX versus CisGem first-line chemotherapy for locally advanced non resectable or metastatic biliary tract cancer (AMEBICA)-PRODIGE 38: Study protocol for a randomized controlled multicenter phase II/III study. Dig. Liver Dis..

[140] Shroff R.T., Javle M.M., Xiao L., Kaseb A.O., Varadhachary G.R., Wolff R.A., Raghav K.P.S., Iwasaki M., Masci P., Ramanathan R.K. (2019). Gemcitabine, Cisplatin, and nab-Paclitaxel for the Treatment of Advanced Biliary Tract Cancers: A Phase 2 Clinical Trial. JAMA Oncol..

[141] Silakit R., Kitirat Y., Thongchot S., Loilome W., Techasen A., Ungarreevittaya P., Khuntikeo N., Yongvanit P., Yang J.H., Kim N.H. (2018). Potential role of HIF-1-responsive microRNA210/HIF3 axis on gemcitabine resistance in cholangiocarcinoma cells. PLoS ONE.

[142] Asukai K., Kawamoto K., Eguchi H., Konno M., Asai A., Iwagami Y., Yamada D., Asaoka T., Noda T., Wada H. (2017). Micro-RNA-130a-3p Regulates Gemcitabine Resistance via PPARG in Cholangiocarcinoma. Ann. Surg. Oncol..

[143] Li Q., Xia X., Ji J., Ma J., Tao L., Mo L., Chen W. (2017). MiR-199a-3p enhances cisplatin sensitivity of cholangiocarcinoma cells by inhibiting mTOR signaling pathway and expression of MDR1. Oncotarget.

[144] Jiao D., Yan Y., Shui S., Wu G., Ren J., Wang Y., Han X. (2017). miR-106b regulates the 5-fluorouracil resistance by targeting Zbtb7a in cholangiocarcinoma. Oncotarget.

[145] Morishita A., Oura K., Tadokoro T., Fujita K., Tani J., Masaki T. (2021). MicroRNAs in the Pathogenesis of Hepatocellular Carcinoma: A Review. Cancers.

[146] Rupaimoole R., Slack F.J. (2017). MicroRNA therapeutics: Towards a new era for the management of cancer and other diseases. Nat. Rev. Drug Discov..

